# A national screening for the prevalence and profile of disability types among Egyptian children aged 6–12 years: a community-based population study

**DOI:** 10.1186/s12889-023-16489-8

**Published:** 2023-08-22

**Authors:** Ammal M. Metwally, Ebtissam M. Salah El-Din, Ghada A. Abdel-Latif, Dina A. Nagi, Lobna A. El Etreby, Ali M. Abdallah, Zeinab Khadr, Randa I. Bassiouni, Ehab R. Abdel Raouf, Amal Elsaied, Alshaimaa A. Elkhatib, Sara F. Sallam, Marwa M. El-Sonbaty, Manal A. Shehata, Nahed A. Elghareeb, Hala Y. Badawy, Doaa E. Ahmed, Nihad A. Ibrahim, Hanaa Emam, Soha M. Abd El Dayem, Asmaa M. Fathy

**Affiliations:** 1 Community Medicine Research Department/ Medical Research and Clinical Studies Institute, National Research Centre (Affiliation ID: 60014618), Dokki, Cairo, Egypt; 2 Child Health Department/ Medical Research and Clinical Studies Institute, National Research Centre (Affiliation ID: 60014618), Dokki, Cairo, Egypt; 3 Clinical Genetics Department/ Human Genetics and Genome Research Institute, National Research Centre (Affiliation ID: 60014618), Dokki, Cairo, Egypt; 4 Quantitative Methods Department, Aswan University, Aswan, Egypt; 5 Department of Statistics, Faculty of Economics and Political Science, Cairo University, Cairo, Egypt; 6 The Social Research Center of the American University in Cairo, Cairo, Egypt; 7 Child With Special Needs Department/ Medical Research and Clinical Studies Institute, National Research Centre (Affiliation ID: 60014618), Dokki, Cairo, Egypt; 8 Prevention of Disability General Directorate, Ministry of Health and Population, Giza, Egypt; 9 Skin and Venereal Diseases Research Department, Medical Research and Clinical Studies Institute, National Research Centre (Affiliation ID: 60014618), Dokki, Cairo, Egypt; 10 Pediatrics Dept. Medical Research and Clinical Studies Institute, National Research Centre, P.O: 12622, Dokki, Cairo Egypt

**Keywords:** Disability, School age children, Children Aged 6–12 Years, Vision, Hearing, Speech, Communication, Mobility—Intellectual impairment, Seizures

## Abstract

**Aim:**

This study aimed to determine the prevalence of disability domains among Egyptian children in the age group of 6–12 years as well as assess their socio-demographic, epidemiological, and perinatal predictors.

**Methods:**

A national population-based cross-sectional household survey targeting 20,324 children from eight governorates was conducted. The screening questionnaire was derived from the WHO ten-question survey tool validated for the identification of disabilities.

**Results:**

The prevalence of children with at least one type of disability was 9.2%. Learning/ comprehension was the most prevalent type (4.2%), followed by speech/communication (3.7%), physical/ mobility and seizures (2.2% for each), intellectual impairment (1.5%), visual (0.7%), and hearing (0.4%). The commonest predictors for disabilities were children who suffered from convulsions or cyanosis after birth and maternal history of any health problem during pregnancy. However, preterm and low birth weight (LBW) babies or being admitted to incubators for more than two days were strong predictors for all disabilities except hearing disability. A history of jaundice after birth significantly carried nearly twice the odds for seizures (AOR = 2.2, 95% CI:1.5–3.4). History of difficult labor was a predictor of intellectual impairment (AOR = 1.5, 95% CI:1.1–2.0). A disabled mother was a strong predictor for all disabilities except seizures, while a disabled father was a predictor for visual and learning/ comprehension disabilities (AOR = 3.9, 95% CI:2.2–7.1 & AOR = 1.6, 95% CI:1.1–2.4 respectively). Meanwhile, both higher maternal and paternal education decreased significantly the odds to have, physical/ mobility and Learning/ comprehension by at least 30%.

**Conclusion:**

The study found a high prevalence of disability among Egyptian children aged 6–12 years. It spotted many modifiable determinants of disability domains. The practice of early screening for disability is encouraged to provide early interventions.

**Supplementary Information:**

The online version contains supplementary material available at 10.1186/s12889-023-16489-8.

## Introduction

According to the World Health Organization, the International Classification of Functioning, Disability, and Health (ICF) outlines disability as an umbrella term, confining impairment, activity, and participation limitations [1]. In other words, disability is part of the human condition that will temporarily or permanently impair all individuals at some point in life, and those who survive old age will experience increasing difficulties in functioning [2]. Globally, it is estimated that one in 20 of those under 15 years of age live with a moderate or severe disability [3]. Living with a disabled child can have profound adverse effects on the entire family as financial costs, physical and emotional demands, time, and logistical complexities [4].

No definite figure was reported denoting the prevalence of disability worldwide. Several factors contribute to reporting the accuracy of the prevalence of disability as reported by a study that reviewed the global disability prevalence and trends [5]. These factors include the exact definition of disability, for which there is no single correct definition of disability, different methodologies of data collection, and variation in the study design. Moreover, this review reported that the nature and severity of disabilities vary greatly and that how disability is measured differs depending on the purpose for measuring it [5]. Most African countries depend on the United Nations (UN) estimates, which reported that about 5% of children in the age group of 0–14 years had one or more disabilities [6]. Although 80% of people with disabilities reside in less developed countries (LDCs), literature concerned with LDC populations with disabilities in general and youth with disabilities is specifically scarce, with Egypt being no exception [7]. Most of the research previously conducted in Egypt focused on specific groups of disabled individuals; namely: blind, mute and deaf/mute, mental disorders, amputation of one or more limbs/ inability to use one or more limbs, paralysis of lower limbs or of all limbs, with small sample size among different age groups [8, 9]. The lack of basic information related to the national estimated levels of disabilities and their risk factors among school-aged children has decreased conducting of intervention studies targeting this group for improving their quality of life. Screening in community surveys is a good method that provides a rapid reflection of this problem among children. Many community surveys have been developed for handicapped children in different parts of the world. The ten questions survey tool provided by the WHO was proposed for this purpose and has been validated for its sensitivity and specificity in many developing countries [10, 11].

The current study aimed at developing a precise and accurate map for disability among school-age children in Egypt. According to the WHO classification, disability domains where children are at greater risk include vision, hearing, speech, mobility, communication/comprehension, learning, intellectual impairments, and seizures [1, 2, 11]. No studies have been published in Egypt detailing the complete set of domains at the national level [12]. To achieve this the current study aimed at determining the magnitude of the prevalence of disability among Egyptian children in the age group of 6–12 years according to the disability domain. The study assessed also the risk factors of the epidemiological, perinatal, socio-demographic characteristics, and socio-economic background.

The findings of our study will help policymakers to prepare for the inclusion of People With Disabilities (PWDs) in mainstream schools and to implement education policies and strategies that respond to the actual needs of children with disabilities. In fact, Egypt has started in the last three decades to move towards the inclusion of PWDs in public education according to UNESCO recommendations for promoting child-friendly learning environments for all children, including those with disabilities [13]. Although most schools have children with a range of disabilities yet, they do not have trained teachers who have specialized in these paths. Another important challenge is the lack of data about different types of disabilities, the definite percentage of disabled children in each grade in different schools and governorates, and the sex prevalence of each disability in different stages of education. So, this study will help in better understanding the potential and significant predictors for the studied disability domains to be targeted, monitored, and accessed by specialized health services. This will help also in allocating resources toward the most required strategies. Some of these strategies include aligning the curricula with the needs of disabled children in different education classes, modifying school buildings to suit the physical condition of PWDs, and improving the quality of education in all special education schools [14].

## Methodology

### Study type

A cross-sectional national community-based prevalence survey with a house-to-house survey was conducted.

### Target group and subject inclusion criteria

The study focused on children any child aged 6 to 12 years who experienced normal milestones for his age as well as any child who met WHO classification of disabilities [1] was also included in the current study whether previously identified as having disability or newly diagnosed during the current study.

### Study design and setting

According to the Central Agency for Public Mobilization and Statistics (CAPMAS) for the 2017 census [15], the geographical classification of Egypt included four urban governorates; Cairo, Alexandria, Port Said, and Suez, nine governorates in Lower Egypt: Damietta, Monufia, Gharbia, Kafr El Sheikh, Dakahlia, Behaira, Sharqi, Ismailia and Qalioubia Governorates and nine governorates in Upper Egypt: Giza, Menya, Beni Suef, Fayoum, Sohag, Assuit, Qena, Luxor and Aswan Governorates. The five Frontier governorates are the Red Sea, New Valley, Matrouh, North Sinai, and South Sinai. The survey was conducted in four stages in 8 Governorates representing these geographic regions of Egypt according to their population density. The first stage included 8 clusters of governorates as follows: one urban (Cairo) represents the greater Cairo region, 3 in Upper Egypt (Fayoum, Assuit, and Aswan) representing the three upper Egypt regions, 3 in Lower Egypt (Damietta, Dakahlia, and Gharbia) representing Delta region and one border -Frontier- (Marsa Matrouh) representing Alexandria Region. In the second stage, a representative sample of cities and local units was selected from each governorate. Using the human development index produced by the UNFPA (2003) each governorate was divided into three categories according to their human development scores, namely; low, medium, and high. One city for urban areas and one local unit for rural areas were selected from each category for each governorate) [15, 16]. In the third stage, the selected cities for urban areas were divided into blocks then one or two blocks were chosen for surveying. For the rural areas, one or two villages were selected from the selected local units. In the last stage, households in the selected city blocks and villages were screened randomly. The sample was allocated to be proportional to the size of large governorates. While governorates with relatively small populations were assigned to arbitrary sample sizes with adjusting weights during the analysis of the data. The survey was conducted over 24 months.

### Sample size

Sample size calculation is based on the estimated prevalence of disability at 5% as indicated globally [3], the level of accuracy was set at 0.0049 (margin of error) and the confidence limit at 95% [17]. The approximate average number of Egyptian children in the age range 6–12 years within each family is 2 [18]. Accordingly, targeting 20,000 children were expected to ensure the provision of estimates of the prevalence of any type of disability as well as for targeting and defining suspicious cases. The sample type was systematic and proportionate to size (pps), for which the selecting sample areas were selected to be proportional to their population according to Egypt's administrative subdivisions, as well as the urban and rural sectors.

### Screening tools

The validated WHO ten questions screening tool (TQS) [11] for disability detection was used during this screening study. This tool was validated to be used for children aged up to 17 years [19, 20]. The sensitivity of TQS in detecting disability was 100% with high false predictive values that would benefit suspected cases of disabilities for more medical attention [11]. The questions used to identify and investigate the following disabilities’ domains: 1) difficulty seeing (in the daytime or night), 2) difficulty hearing, 3) comprehension (unable to understand orders), 4) movement (weakness or stiffness in the arm(s)/leg(s)), 5) Seizure (have fits, rigidness or loss of consciousness), 6) learning (unable to do something like other children his/her age), 7) Speech (no speech), 8) communication (unclear speech) and 9) Intellectual impairment (appeared mentally backward/dull or slow). The categorization of disabilities was based on the WHO, International Classification of Functioning, Disability, and Health, Version for Children and Youth [21, 22] to include seven categories for which learning and comprehension were included in the same category, Speech, and communication were also included in another category. Developmental milestones (for any disability in sitting, standing, or walking), were not included or discussed in this survey being verified by other more validated tools and were published in a separate research [23].

The first part of the household questionnaire collected information on the WHO ten questions screening (TQS) for the detection of common disabilities. The second part of the questionnaire collected information on housing characteristics e.g. the number of rooms, the source of water, and ownership of a variety of consumer goods. For the third part of the questionnaire, questions included demographic information about the members of the households.

Discovered children with special needs were referred to specialized physicians in the health centers to be managed.

### Implementation of fieldwork

The implementation of this screening was carried out at the household level. The questionnaire was directed to parents and caregivers of children aged 6- 12 years through face-to-face interviews. The three parts of the developed questionnaire were fulfilled. The fieldwork was carried out by pre-trained professional field surveyors.

The survey was conducted under the supervision of a team including members from the Cairo Demographic Center (CDC), the National Research Centre (NRC), and the Disability Reduction Department of the Egyptian Ministry of Health and Population (MOHP). The team included epidemiologists, and public health specialists, in addition to specialists from several departments at NRC as the Children with special needs department, clinical genetics, and the Behavioral and developmental pediatric department.

### Statistical analysis

Frequencies and proportions as well as means ± standard deviation (SD) were used to describe categorical and continuous variables respectively. Comparisons between groups were done using odds ratios (OR) and 95% confidence intervals (CI) were calculated in comparison between children having different types of disabilities and healthy children. Logistic regression analysis was done to assess the contribution of each independent variable to predict the odds of developing any domain of disability and those without this disability based on the values of the independent variables (risk factors for disability) [24]. Results were presented in terms of crude odds ratio (COR) and adjusted odds ratio (AOR) in a univariate and multivariate analysis respectively. A significant association was considered if the 95% CI did not include the value of 1.0. All statistical analyses were carried out using Statistical Package for Social Sciences (SPSS) software version 22.0 software (IBM SPSS Statistics for Windows, Version 22.0. Armonk, NY: IBM Corp.). P-values less than 0.05 were considered statistically significant.

## Results

The study population characteristics were shown in Table 1. The total number of surveyed children aged 6–12 years was 20,324. Boys represented 51.0% of the whole sample versus 49.0% of girls. Most children were going to school (95.7%), belonging to rural localities (52.2%), and were equally distributed among social classes.

**Table 1:** Characteristics of the study population

Socio-demographic characteristics	Surveyed children (20,324)	
	**n**	**Column%**
**Locality (Urban/ Rural)**		
Urban	9715	47.8
Rural	10,609	52.2
**Social class**		
Low	6662	32.8
Middle	6794	33.4
High	6868	33.8
**Geographical Distribution**		
Cities	3655	18.0
Lower Egypt	7971	39.2
Upper Egypt	6639	32.7
Frontier	2059	10.1
**Sex**		
Boys	10,361	51.0
Girls	9963	49.0
**Age category**		
**Current child age**		
Mean age ± SD	8.8 ± 1.7	
**Current mother age**		
Mean age ± SD	34.6 ± 5.9	
**Mother age at giving birth**		
Mean age ± SD	25.8 ± 5.7	
< 18	1135	5.6
18 to < 35	17,554	86.4
≥ 35	1440	7.1
**Children going to school**	19,460	95.7
**Mother Education**		
Illiterate/ Read & write/ Primary/ Prep	7896	38.9
High School & technical/ above intermediate	9177	45.2
University or higher	3052	15.0
**Father Education**		
Illiterate/ Read & write/ Primary/ Prep	7537	37.1
High School & technical / Above intermediate	8523	41.9
University or higher	3213	15.8
**Mother work**		
Employed	3038	14.9
Unemployed	17,093	84.1
**Mean of HH members ± SD**	5.3 ± 1.3	
**Presence of mothers or fathers**		
No father at home	1044	5.1
No mother at home	189	0.9
**Twin child**	799	3.9
**Disabled mothers #**	208	1.0
**Disabled fathers #**	369	1.8
**Perinatal problems**		
Premature children (< 37 weeks gestation)	184	0.9
Low birth weight (< 2500 mg)	771	3.8
Children suffered from jaundice after birth	4848	23.9
Children suffered from bluish discoloration after birth (Cyanosis)	233	1.1
Children suffered from any convulsions	320	1.6
Children were admitted to an incubator for more than two days	1308	6.4
Mothers had any health problems during pregnancy*	1270	6.2
History of difficult labor**	2657	13.1

^#^ disabled mothers or fathers: physically or mentally disabled; Hearing, Vision, Mental, Movement, Speech[19, 20]

^*^Mothers had any pregnancy complications as iron deficiency anemia, gestational diabetes, hypertension, infection, anxiety or depression [25]

^**^Difficult labor refers to prolongation in the duration of labor, typically in the first stage of labor. It can be an important contributor to maternal and perinatal mortality and morbidity if it remains unrecognized or untreated [26]

Regarding the distribution of the mothers’ ages at birth, the majority were in the age range of 18—< 35 years (86.4%). Most mothers and fathers had high school or technical and above intermediate education (45.2% and 41.9% respectively). Most of the mothers were housewives and unemployed. Houses without mothers were 0.9% versus 5.1% headed by mothers without fathers. Mothers and fathers with disabilities were 1.0% and 1.8% respectively. Twin children represent 3.9% of the surveyed children. Neonatal jaundice was the most prevalent perinatal problem (23.9%).

**Fig. 1:**
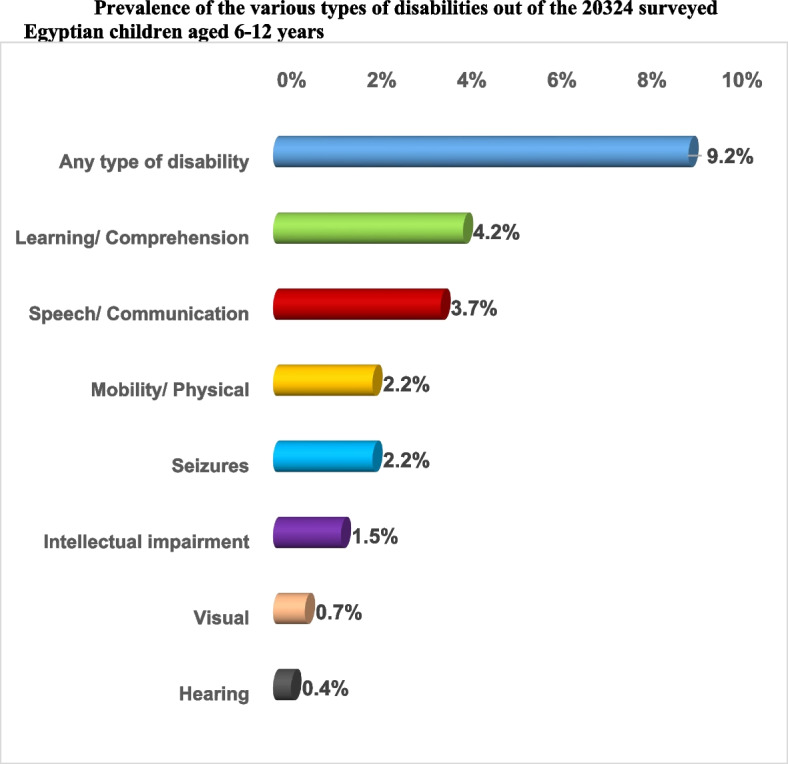
Prevalence of the various types of disabilities out of the 20,324 surveyed Egyptian children aged 6–12 years

The prevalence of the various types of disabilities among children aged 6–12 years was illustrated in Fig. 1. The prevalence of at least one type of disability among children aged 6–12 years was 9.2%. The prevalence of disabilities was as follows: learning/ comprehension (4.2%), speech/ communication (3.7%), mobility/ physical & seizures (2.2% each), intellectual impairment (1.5%), visual (0.7%), and the least were listening (0.4%).

Table 2 shows the odds of having handicapping disabilities. Concerning the sociodemographic factors, the odds for the presence of at least one disability in cities was nearly twice that in frontier, upper, and lower Egypt governorates with the prevalence of 13.0%, 7.6%, 8.1%, and 8.7% respectively. The odds were significantly 1.3 times higher among urban than rural communities (prevalence 10.3% vs. 8.1%) and among low social class than high class (9.3% vs. 8.2%).

**Table 2:** Sociodemographic and Epidemiological characteristics of the studied population with respect to disabilities

**Socio-demographic and epidemiological characteristics**	**Children with at least one disability (total children with disabilities)** **n = 1864**		**Healthy children** **n = 18,460**		**COR (CI)** §
	**n**	**Raw %**	**n**	**Raw %**	
**Locality (Urban/ Rural)**					
Urban	1001	10.3	8714	89.7	urban vs. rural: 1.3 (1.2–1.4)**
Rural	863	8.1	9746	91.9	
**Social class**					
Low	618	9.3	6044	90.7	low vs. middle: 0.9 (0.8–1.0)
Middle	680	10.0	6114	90.0	middle vs. high: 1.2 (1.1–1.4)**
High	566	8.2	6302	91.8	low vs. high: 1.1 (1.0–1.3)*
**Geographical Distribution**					
Cities	476	13.0	3179	87.0	cities vs. lower: 1.6 (1.4–1.8)**
Lower Egypt	696	8.7	7275	91.3	lower vs. frontiers: 1.2 (0.9–1.4)
Upper Egypt	535	8.1	6104	91.9	cities. vs upper: 1.7 (1.5–1.9)**
Frontier	157	7.6	1902	92.4	cities vs. frontiers: 1.8 (1.5–2.2)**
**Sex**					
Boys	1139	11.0	9222	89.0	boys vs. girls: 1.6 (1.4–1.7)**
Girls	725	7.3	9238	92.7	
**Child go to school**					
Do not go to school	203	23.7	655	76.3	child not go to school vs. go to school: 3.3 (2.8–3.9)**
Go to school	1661	8.5	17,799	91.5	
**Mother age at giving birth**					
< 18	90	7.9	1045	92.1	≥ 35 vs. < 18: 1.4 (1.1–1.9)*
18 to < 35	1585	9.0	15,969	91.0	< 18 vs. 18- < 35: 0.9 (0.7–1.1)
≥ 35	157	10.9	1283	89.1	≥ 35 vs. 18- < 35: 1.2 (1.0–1.5)*
**Mothers Education**					
Illiterate/ Read & write/ Primary/ Prep (1)	806	10.2	7090	89.8	(3) vs. (1): 0.6 (0.5–0.7)**
High School & technical/ above intermediate (2)	830	9.0	8347	91.0	(2) vs. (1): 0.87 (0.78–0.96)*
University or higher (3)	196	6.4	2856	93.6	(3) vs. (2): 0.7 (0.6–0.8)**
**Fathers Education**					
Illiterate/ Read & write/ Primary/ Prep (1)	811	10.8	6726	89.2	(3) vs. (1): 0.5 (0.4–0.6)**
High School & technical/ above intermediate (2)	725	8.5	7798	91.5	(2) vs. (1): 0.8 (0.7–0.9)**
University or higher (3)	192	6.0	3021	94.0	(3) vs. (2): 0.7 (0.6–0.8)**
**Mothers work**					
Unemployed (1)	1601	9.4	15,492	90.6	(1) vs. (2): 1.3 (1.1–1.5)**
Employed (2)	231	7.6	2807	92.4	
**Presence of mother or father**					
No father at home	135	12.9	909	87.1	no father vs. father at home: 1.5 (1.3–1.8)** no mother vs. mother at home: 2.0 (1.4–3.0)** no mothers versus no fathers: 1.4 (0.9–2.1)
No mother at home	32	16.9	157	83.1	
**Twin child**					
Twins	83	10.4	716	89.6	twin vs. no twin: 1.2 (0.9–1.5)
No twins	1781	9.1	17,744	90.9	
**Disabled mother**					
Mothers with disability	52	25.0	156	75.0	disabled mother vs. no disability: 3.4 (2.5–4.7)**
No disability	1780	8.9	18,150	91.1	
**Disabled father**					
Father with disability	72	19.5	297	80.5	disabled father vs. no disability: 2.5 (1.9–3.3)**
No disability	1657	8.8	17,254	91.2	

^*^ = *p*-value significant at < 0.05

^**^ = *p*-value highly sig at < 0.01

^§^ = The first variable written in the column is considered as the reference (risky or protective variable)

*CI* Confidence Interval, *COR* Crude Odds Ratio

Concerning the epidemiological factors, the boys were nearly one and a half times more likely than girls to be diagnosed with any disability (COR = 1.6, 95% CI: 1.4–1.7). Children not going to school were the most likely to be diagnosed with any disability with a prevalence of 23.7% which was significantly three times higher than the prevalence among children going to school (COR = 3.3, 95% CI: 2.8–3.9).

Children of mothers aged > 35 years at giving birth had significantly higher odds of occurrence of any type of disability than those of mothers aged < 18 or 18- < 35 years at giving birth (COR = 1.4 & 1.2 respectively). Meanwhile, living without mothers and/or fathers in homes increased the odds of having disabilities by two times (COR = 2.0, 95% CI: 1.4–3.0) and one and a half times respectively (COR = 1.5, 95% CI: 1.3–1.8) with higher odds than when living without mothers. Unemployment of mothers significantly affected the odds of having a child disability by more than one time (COR = 1.3, 95% CI: 1.1–1.5).

Children with mothers or fathers who had higher education were significantly less likely to have any type of disability with the least odds for the mothers and fathers who had a college or higher education level (COR = 0.7, 95% CI: 0.6–0.8 each). Disabled mothers and/or fathers carried the odds to have a disabled child more than three times (COR = 3.4, 95% CI: 2.5–4.7) and two & half times respectively (COR = 2.5, 95% CI: 1.9–3.3).

**Fig. 2:**
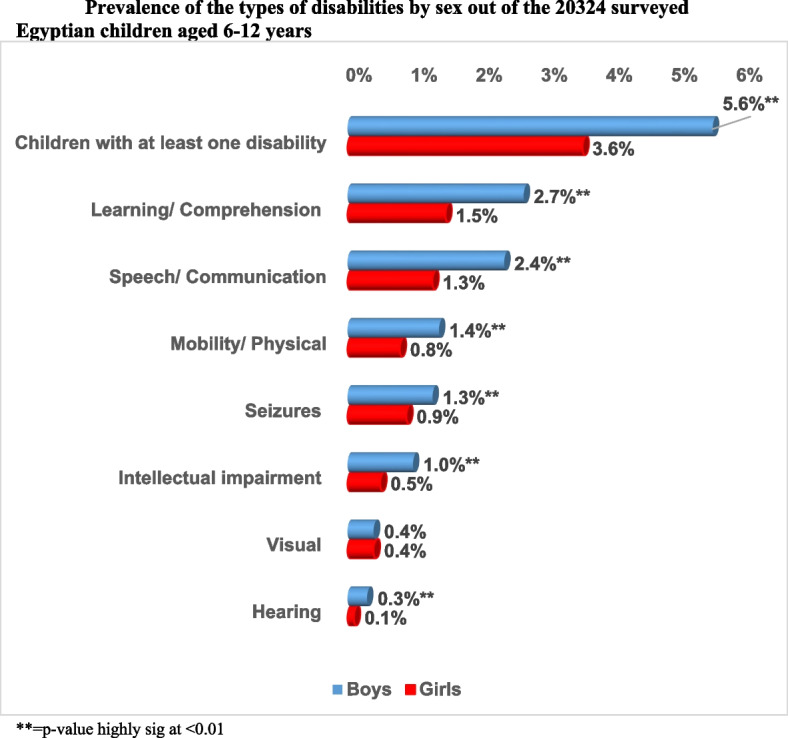
Prevalence of the types of disabilities by sex out of the 20,324 surveyed Egyptian children aged 6–12 years. ** = *p*-value highly sig at < 0.01

Among the surveyed children aged 6–12 years, the prevalence of boys with at least one disability was higher than girls (5.6% Vs. 3.6% respectively). Most boys and girls had learning/ comprehension disabilities (2.7% and 1.5%, respectively) followed by speech disabilities (2.4% and 1.3% respectively). Visual disability was seen equally among girls and boys (0.4% each), whereas hearing (0.3%), mobility/ physical (1.4%), intellectual impairment (1.0%), and seizures (1.3%) were seen more among boys. The pattern of distribution of disability types according to sex was significant for all disability domains except for visual disability (Fig. 2).

**Fig. 3:**
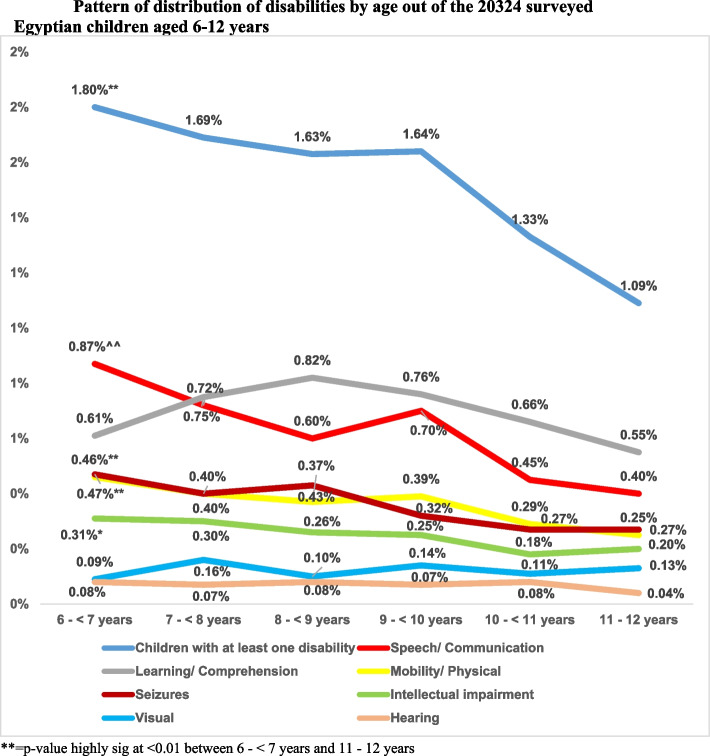
Pattern of distribution of disabilities by age out of the 20,324 surveyed Egyptian children aged 6–12 years. ** = *p*-value highly sig at < 0.01 between 6—< 7 years and 11—12 years

The pattern of distribution of disabilities out of the 20,324 surveyed children was studied at one year gap in Fig. 3. The prevalence of children with at least one disability was highest for children aged 6- < 7 years (1.8%). It showed a steady decrease till the age of 9- < 10 years then a sudden decrease thereafter to reach 1.09% at the age group 11–12 years. The highest prevalence of speech & mobility/ physical disabilities, intellectual impairment and seizures was found among children aged 6- < 7 years (0.87%, 0.47%, 0.31% and 0.46 respectively). The highest prevalence of learning/ comprehension disability was found among children aged 8- < 9 years (0.82%). While the prevalence of hearing disability was highest for children aged 8- < 9 years and 10- < 11 years (0.08% each). In addition, the highest prevalence of visual disability was found among children aged 11- < 12 years (0.13%).

The percentage of children having one disability was 6.0% versus 3.2% with multiple disabilities. The prevalence rate of the types of disabilities of the surveyed children by the number of disabilities is shown in Table 3. The prevalence of learning/ comprehension disability was the highest (4.2%), followed by speech disability (3.7%). Hearing disability was the least prevalent disability (0.4%). seizures were most probable to be found as combined disabilities mainly among boys than girls with nearly twice the odds (COR = 1.8, 95% CI: 1.2–3.7).

**Table 3:** Distribution of the number of disabilities by types of disabilities of the surveyed children aged 6 – 12 years in Egypt

Type of Disability	Multiple disabilities		One disability		Total		Boys Vs. Girls
**Total number surveyed**	**648 (3.2)**		**1216 (6.0)**		**20,324**		
	**n**	**%**	**n**	**%**	**n**	**%**	**COR (CI)**
**Visual (total)**	49	7.6	101	8.3	150	0.7	
Boys	25	3.9	48	3.9	73	0.3	1.2 (0.6–2.3)
Girls	24	3.7	53	4.4	77	0.4	
**Hearing (total)**	52	8.0	34	2.8	86	0.4	
Boys	40	6.2	23	1.9	63	0.3	1.1 (0.6–2.3)
Girls	12	1.9	11	0.9	23	0.1	
**Speech/ Communication (total)**	489	75.5	271	22.3	760	3.7	
Boys	315	48.6	172	14.1	487	2.4	1.0 (0.8–1.4)
Girls	174	26.9	99	8.1	273	1.3	
**Mobility/ Physical (total)**	315	48.6	129	10.6	444	2.2	
Boys	207	31.9	77	6.3	284	1.4	1.3 (0.8–2.0)
Girls	108	16.7	52	4.3	160	0.8	
**Learning/ Comprehension (total)**	494	76.2	352	28.9	846	4.2	
Boys	317	48.9	221	18.2	538	2.7	1.1 (0.8–1.4)
Girls	177	27.3	131	10.8	308	1.5	
**Intellectual impairment (total)**	276	42.6	27	2.2	303	1.5	
Boys	193	29.8	15	1.2	208	1.0	1.9 (0.8–4.1)
Girls	83	12.8	12	1.0	95	0.5	
**Seizures (total)**	137	21.1	302	24.8	439	2.2	
Boys	92	14.2	161	13.2	253	1.3	1.8 (1.2–2.7)**
Girls	45	6.9	141	11.6	186	0.9	

^*^ = *p*-value significant at < 0.05, ** = *p*-value highly sig at < 0.01

*CI* Confidence Interval, *COR* Crude Odds Ratio

Table 4 shows the multivariate logistic regression model for exploring the predictors of disabilities among children aged 6 – 12 years. The odds of having several disabilities were significantly higher among male children than among females for mainly hearing (AOR = 2.6, 95% CI: 1.6–4.3), speech, mobility/ physical & learning/ comprehension disabilities, and intellectual impairment. Going to school significantly decreased the odds to have all disabilities.

**Table 4:** Multiple Logistic regression model for prediction of diagnosis. (Each type of disability vs healthy without this type of disability)

	**Visual**		**Hearing**		**Speech/ Communication**		**Mobility/ Physical**		**Learning/ Comprehension**		**Intellectual impairment**		**Seizures**	
	**AOR**	**CI**	**AOR**	**CI**	**AOR**	**CI**	**AOR**	**CI**	**AOR**	**CI**	**AOR**	**CI**	**AOR**	**CI**
**Epidemiological and Socioeconomic factors**														
Sex (male is the base)	0.9	0.7	2.6**	1.6	1.7**	1.5	1.7**	1.4	1.7**	1.5	2.0**	1.6	1.1	0.8
		1.3		4.3		2.0		2.1		2.0		2.6		1.6
Locality (urban – rural) urban is the base	1.1	0.7	1.1	0.6	1.1	0.9	1.2	0.9	1.2	0.9	1.6**	1.1	0.8	0.5
		1.8		1.9		1.3		1.5		1.4		2.1		1.3
**Social level**														
High to low **(High is the base)**	1.3	0.9	1.3	0.8	1.1	0.9	0.9	0.7	0.9	0.8	0.9	0.7	0.5**	0.4
		1.9		2.1		1.3		1.2		1.1		1.2		0.8
Middle to High (M**iddle is the base)**	0.7	0.4	0.7	0.4	1.2	0.9	2.1**	1.7	1.3**	1.1	1.5*	1.1	0.5**	0.3
		1.0		1.3		1.4		2.7		1.6		2.1		0.7
**Geographical distribution: (Lower, Upper, and Frontiers) are the base**														
Lower to cities	1.2	0.7	1.8	0.9	0.8	0.6	0.8	0.5	0.5**	0.4	1.1	0.8	0.5	0.2
		2.0		3.6		1.1		1.1		0.7		1.7		1.1
Upper to cities	0.5**	0.3	0.5	0.2	0.6**	0.5	0.5**	0.4	0.8*	0.6	0.5**	0.3	0.4**	0.2
		0.8		1.1		0.8		0.7		0.9		0.7		0.7
Frontiers to cities	0.5**	0.3	0.8	0.4	0.8*	0.6	0.6**	0.5	0.6**	0.4	0.7*	0.5	0.8	0.4
		0.8		1.7		0.9		0.9		0.7		0.9		1.5
Children go to school (base is going to school)	0.3	0.2	0.2**	0.1	0.2**	0.1	0.15**	0.10	0.3**	0.2	0.08**	0.06	0.5	0.2
		0.5		0.3		0.2		0.19		0.4		0.11		1.0
**Maternal education: University and above is the base**														
University and above to less education	0.8	0.4	0.4	0.2	0.7*	0.5	0.6*	0.4	0.6**	0.5	0.8	0.5	1.2	0.7
		1.5		1.2		0.9		0.9		0.9		1.2		2.4
**Paternal education: Less education is the base**														
University and above to less education	1.3	0.7	1.3	0.6	0.8	0.6	0.5**	0.4	0.5**	0.4	0.4**	0.2	0.6	0.3
		2.2		2.8		1.0		0.8		0.7		0.7		1.2
**Disability of Mothers or Fathers**														
Disabled mother is the base	5.7**	3.0	2.6	0.7	1.9*	1.1	2.1*	1.1	2.3**	1.4	1.8	0.8	0.5	0.1
		10.7		9.2		3.2		4.0		3.6		4.2		3.6
Disabled father is the base	3.9**	2.2	1.3	0.4	0.9	0.5	0.8	0.4	1.6*	1.1	0.5	0.2	0.8	0.2
		7.1		4.5		1.5		1.7		2.4		1.4		3.3
**Maternal problems are the base**														
Mothers had any health problems during pregnancy	2.4**	1.5	1.3	0.5	1.3	0.9	1.5*	1.1	1.6**	1.2	1.2	0.8	1.3	0.7
		4.1		3.0		1.7		2.1		2.0		1.8		2.4
Difficult labor	0.8	0.5	0.9	0.4	1.1	0.9	1.3*	1.0	1.1	0.9	1.5*	1.1	0.9	0.5
		1.4		1.7		1.4		1.7		1.4		2.0		1.4
**Perinatal problems are the base**														
Preterm babies with baby’s weight is less than 2.5 kg at birth (LBW)	1.9*	1.0	0.9	0.3	1.9**	1.4	1.9**	1.3	2.0**	1.5	2.3**	1.5	2.6**	1.5
		3.4		2.7		2.5		2.8		2.6		3.4		4.6
Children suffered from jaundice after birth	1.2	0.8	0.9	0.5	1.2	0.9	1.1	0.9	1.2*	1.0	1.3	0.9	2.2**	1.5
		1.7		1.6		1.4		1.4		1.4		1.7		3.4
Children suffered from cyanosis after birth	4.3**	2.0	4.1*	1.4	2.4**	1.6	2.4**	1.4	2.5**	1.7	1.6	0.9	7.7**	3.7
		9.2		12.0		3.7		3.9		3.8		3.0		16.3
Children suffered from any convulsions after birth	1.4	0.6	3.6**	1.4	3.2**	2.2	3.1**	2.0	2.1**	1.5	4.1**	2.5	1.9 × 10^11^	0.0
		3.2		9.1		4.5		4.8		3.1		6.5		-
Child was admitted to an incubator for more than two days	1.6	0.9	0.8	0.3	1.7**	1.3	1.5*	1.1	1.5**	1.2	2.1**	1.5	2.0*	1.2
		2.7		2.1		2.2		2.1		2.0		3.0		3.3
Constant	0.0**				0.2**		0.1**		0.1**		0.0**		0.0**	

^*^ = *p*-value significant at < 0.05

** = *p*-value highly sig at < 0.01

*CI* Confidence Interval, *AOR* Adjusted Odds Ratio

Variable(s) entered in model 1: age, sex, locality, social class, geographical distribution, maternal age at birth, child go to school, mother education, father education, maternal work status, mother disability, father disability, mother problem during pregnancy, difficult labor, child born less than 7 m, preterm babies with baby weight less than 2.5 kg at birth, child suffer from jaundice after birth, child suffer from bluish discoloration after birth, child suffer from convulsions after birth, child kept in incubator for more than two days

Belonging to the middle social class was associated with higher odds of having mobility/ physical & learning/ comprehension disabilities, and intellectual impairment with a varied range of odds for having a disability which was highest for mobility/ physical disability (AOR = 2.1, 95% CI: 1.7–2.7). Both higher maternal and paternal education decreased significantly the odds to have, physical/ mobility and Learning/ comprehension by at least 30%. Whereas, higher maternal education decreased the odds to have speech/communication by 30% (AOR = 0.7, 95% CI: 0.5–0.9), higher paternal education decreased the odds of having intellectual impairment by 60% (AOR = 0.4, 95% CI: 0.2–0.7). Living in frontier governorates and upper Egypt significantly decreased the odds of having all disabilities than living in cities. Whereas living in lower Egypt decreased significantly only the odds of having learning/ comprehension disabilities (AOR = 0.5, 95% CI: 0.4–0.7) than living in cities. Living in urban communities carried significantly higher odds for only intellectual impairment disabilities than living in rural communities (AOR = 1.6, 95% CI: 1.1–2.1).

The strong predictors for all disabilities were in order: neonatal history of convulsions or cyanosis after birth and mothers having a history of any health problem during pregnancy. Whereas preterm children with LBW or kept in incubators for more than two days were strong predictors for all disabilities except hearing disability. In addition, a history of jaundice after birth significantly carried nearly twice the odds for seizures (AOR = 2.2, 95% CI: 1.5–3.4). History of difficult labor was a significant predictor for intellectual impairment (AOR = 1.5, 95% CI: 1.1–2.0).

Disabled mother was a strong predictor for all disabilities except hearing, intellectual impairment, and seizures. While children of disabled fathers were likely to have visual, and learning/ comprehension disabilities (AOR = 3.9, 95% CI: 2.2–7.1 & AOR = 1.6, 95% CI: 1.1–2.4 respectively).

## Discussion

Detecting the magnitude of disabilities and their types among Egyptian children will guarantee their inclusion in decision-making by ensuring they are counted, consulted, and considered in future health planning. This survey aimed at screening the phenomenon in a large household nation-representative sample to define cases of disabilities.

The survey revealed a 9.2% prevalence of children aged 6–12 years with at least one type of disability which is slightly higher (8.8%) than an Egyptian study that was done in four governorates of Egypt [12]. The Global Burden of Disease estimates childhood disability prevalence to be 95 million (5.1%) children [27]. While UNICEF's global disability prevalence estimates indicated that the Middle East region has one of the highest prevalence rates of children with disabilities between the ages of 5 and 17 by 16.9% [28]. While significant variation in prevalence estimates was found in a study of children in 16 developing countries, the percentages of children who were positive for at least one disability ranged from 3.1%—45.2% [29]. Meanwhile, 7.3% (CI 6.9, 7.7) of UK children were reported as disabled according to the Disability Discrimination Act (DDA) definition [30] and 12% among Indian children aged 5–14 years [31]. The increased prevalence in this study, as in others developing countries, may be attributed to the high consanguineous marriage, high maternal or paternal ages, illiteracy, communicable diseases, and high rates of accidents in Egypt.

In this study, boys were nearly one and half times more likely than girls to be diagnosed with any disability (OR = 1.6, 95% CI: 1.4–1.7), for hearing (OR = 0.4), speech, mobility/ physical & learning/ comprehension disabilities (OR = 0.6 each) and intellectual impairment (OR = 0.5). That is consistent with WHO & World Bank reports, as males were reported to have twice the prevalence of any disabilities than females. However, there is some variation depending on disability type and context [32].

The prevalence of learning/ comprehension disability among children was (4.2%), which is close to the prevalence rate of childhood learning disability reported in India of 3% [33]. While it was 7.66% in the USA [34]. That increase may be due to increasing the number of children going to school in the USA than in Egypt. The prevalence of disabilities was higher among middle and low social class than high-class families (10.0% and 9.3% respectively vs. 8.2%), which is consistent with results of USA screening, as children from families with income below the federal poverty level had a higher prevalence of disabilities [34]. Meanwhile, living without mothers and/or fathers in homes increased the odds of having disabilities by significantly two times (OR = 2.0, 95% CI: 1.4–3.0) and one and half times respectively (OR = 1.5, 95% CI: 1.3–1.8) with higher odds than when living without mothers. This was consistent with the results of a recent national survey that was done in Egypt to detect the prevalence of developmental delays among children up to 12 years [23], indicating a high link between developmental delays and disabilities. This finding justifies the important role of parent–child interactions in improving the developmental, intellectual, and social skills of children [35, 36] and highlights the influence of interaction between different sociodemographic factors on development and disabilities [37, 38].

The prevalence of disabilities was significantly 1.3 times higher among urban than rural communities (10.3% vs. 8.1%). Meanwhile, urban children were more likely to have intellectual impairment than rural children due to a number of factors including a greater risk of accidents and injuries, in addition to the relative social isolation of urban children due to being left at home by working mothers or more conservative lifestyle of families in urban areas.

Going to school significantly decreased the odds to have all disabilities [27]. In this study, children who didn’t go to school were most likely to be diagnosed with any disability with a prevalence of 23.7% which was significantly three times higher than the prevalence among children going to school.

Disabled mothers and/or fathers have significantly more than three times (COR = 3.4, 95% CI: 2.5–4.7) and two & half times respectively (COR = 2.5, 95% CI: 1.9–3.3) the odds to have a disabled child. Whereas being twins had an influence on the probability of disability by more than one time. This was in accordance with the Egyptian national survey for developmental delay among children [23]. This may be due to the genetic predisposition triggered by environmental factors such as trauma, infection, and stress [39–41].

Children with mothers or fathers who had higher education were significantly less likely to have any type of disability with the least odds for the mothers and fathers who had a college or greater education level. The same odds were found in a study that was done in Saudi Arabia claiming that low maternal educational level influenced the risk of disability mainly the visual one [42]. Low education level of parents is associated with decreased awareness of healthy developmental growth, and ways to improve children's intellectual development [27]. The present study found that unemployed mothers were significantly affecting the odds of having a child disability by more than one time (COR = 1.3, 95% CI: 1.1–1.5). Nomaguchi's study reported that early maternal employment had a consequential effect on cognitive and behavioral development [43].

Age is linked significantly with functional difficulties; as age increases, the prevalence of disability increases [34]. In this study, the distribution of disabilities was studied from 6 to 12 years of age. It was found that the prevalence of hearing disability was highest among children aged 10- < 11 years (0.08%). In addition, the highest prevalence of visual disability was found among children aged 11- < 12 years (0.13%). Such a pattern was consistent with the findings of the Egyptian national survey for developmental delays among children aged 1–12 years [23] indicating the importance of early management of developmental delays before deteriorating into disabilities. Growth and aging cause a substantial increase in vision-related disability [44]. The prevalence of multiple disabilities among children was higher than that of a single disability with nearly two times more than a single disability (COR = 1.9 and 1.8 respectively). The possible causes were that the gene or enzyme defects that cause disability usually cause multisystem affection, and produce multiple disabilities in the child [41, 44] or that early developmental delays cause disabilities if neglected [21] or manifest as autism [45].

Multiple predictors can influence disability prevalence, type, and severity. They vary across countries, including trends in health conditions, genetic factors, environmental factors, road traffic crashes, natural disasters, conflict, and diet [28]. Perinatal problems were reported by this study as risk factors for disabilities in school children. In the present study, convulsions or cyanosis after birth and any health problem during pregnancy, fetal prematurity, low birth weight, distress, and asphyxia increased the risk for disability. Preterm children with LBW or kept in an incubator for more than two days were strong predictors for all disabilities except hearing disability, as the most important outcomes of low birth weight (LBW) infants include moderate to severe neurological impairment and lifelong neurodevelopmental disability [46].

In this study, preterm LBW children acted as a risk predictor for nearly all the studied disabilities’ domains. A preterm baby is usually accompanied by a pessimistic perception of survival and further long-life disability [47].

Newborn jaundice is a common presentation in the first week of life. In its severe form, it can lead to both mortality and long-term disability [48]. In the present study, a history of jaundice after birth significantly carried nearly twice the odds for seizures (AOR = 2.2, 95% CI: 1.5–3.4).

In agreement with Ayenew, et al., 2021, who found that difficult labor is a major contributing factor in the physical disability of the baby, the current study reported difficult labor as a predictor for mobility/ physical disability and intellectual impairment [49]. Fortunately, the majority of risk factors for different disabilities domains are preventable ones. Some previous Egyptian studies highlighted the importance of empowering women with health education and improving care-seeking behavior which has a great impact on birth outcomes [50–52]. Moreover, the influence of the paternal, and maternal parameters as well as the nutritional supplementation on child growth and development is well evident in many Egyptian studies [53–56], suggesting the success of early prevention for promoting children's health. A population-based birth cohort study from sub-Saharan Africa reported significant protective effects of maternal education, birth weight, and socioeconomic status on developmental outcomes. Moreover, a population-based birth cohort study from sub-Saharan Africa reported significant protective effects of maternal education, birth weight, and socioeconomic status on developmental outcomes [57].

Millions of disabled children around the world continue to be left behind, despite the near-universal ratification of the Convention on the Rights of the Child, set by the Sustainable Development Goals. This neglect is often the result of limited data. When absent from official statistics, children and adults with disabilities remain politically and socially ‘invisible’, increasing their marginalization. National screening and identifying developmental disabilities, early in life allows children and their families to get the help they need. Avoidance of the preventable causes of disabilities is a must. Including children with disabilities in all aspects of life is a priority. Every child, everywhere, has something to offer. His or her energies, talents, and ideas can make a positive difference to families, communities, and the world.

### Strengths of the study

Our study is a community-based population one, representing all geographical areas with a very large sample size with high confidence and accuracy levels. This study was the first to highlight not only the national prevalence of disabilities but focused also the need for community-driven data to detect the preventable types of disabilities. The study contributed to Sustainable Development Goal 10 and the United Nations Convention on the Rights of Persons with Disabilities for not leaving any child behind agenda. Accordingly, the study provided essential data upon which more inclusive strategies and practices can be developed to support the educational and social development of children with functional difficulties.

### Limitation

As this study was screening and had to be completed in a short time of about 15-20min, it was limited in studying the following factors: environmental factors, the influence of the nutritional factors although well documented to affect development in many Egyptian studies both early in life due to exclusive breastfeeding [58] and during proper weaning [59]. A thorough investigation of the nutritional pattern and environmental factors is known to take a long time.

## Conclusion and recommendation

The current study is the first national study estimating the prevalence of disabilities among children aged 6–12 years in Egypt, 9.2% of the investigated children had at least one disability. The most prevalent delay was Learning/ comprehension, and the least was hearing. Children who suffer from convulsions after birth or cyanosis after birth and if mothers had a history of any health problem during pregnancy significantly carry the highest odds for all disabilities. Prevention of disabilities is from national priority. Early screening for a disability should be encouraged to allow early interventions. The outcomes of this study can contribute to the body of evidence that supports and eventually enhances community education, disabilities screening, and diagnostic efforts to improve early identification, and proper management of disabilities in children.

### Supplementary Information

**Additional file 1: S1.** Raw data Disability 6-12 years.

## Data Availability

The datasets used and analyzed during the current study are fully available without restriction. They are included in this published article as supplementary information files.

## Abbreviations

AOR: Adjusted Odds Ratio
CDC: Cairo Demographic Center
CI: Confidence Interval
DDA: Disability Discrimination Act
HH: Household
LBW: Low birth weight
MOHP: Ministry of Health and Population
NRC: National Research Centre
PWDs: People With Disabilities
SPSS: Statistical package of social sciences
UK: United Kingdom
USA: United states of America
WHO: World health organization
